# Identification of candidate neoantigens produced by fusion transcripts in human osteosarcomas

**DOI:** 10.1038/s41598-018-36840-z

**Published:** 2019-01-23

**Authors:** Susan K. Rathe, Flavia E. Popescu, James E. Johnson, Adrienne L. Watson, Tracy A. Marko, Branden S. Moriarity, John R. Ohlfest, David A. Largaespada

**Affiliations:** 1 Masonic Cancer Center, University of Minnesota, Minneapolis, MN USA; 2 Supercomputing Institute, University of Minnesota, Minneapolis, MN USA; 3 Department of Genetics, Cell Biology and Development, University of Minnesota, Minneapolis, MN USA; 4 Center for Genome Engineering, University of Minnesota, Minneapolis, MN USA; 5 Department of Pediatrics, University of Minnesota, Minneapolis, MN USA

## Abstract

Osteosarcomas are characterized by highly disrupted genomes. Although osteosarcomas lack common fusions, we find evidence of many tumour specific gene-gene fusion transcripts, likely due to chromosomal rearrangements and expression of transcription-induced chimeras. Most of the fusions result in out-of-frame transcripts, potentially capable of producing long novel protein sequences and a plethora of neoantigens. To identify fusions, we explored RNA-sequencing data to obtain detailed knowledge of transcribed fusions, by creating a novel program to compare fusions identified by deFuse to *de novo* transcripts generated by Trinity. This allowed us to confirm the deFuse results and identify unusual splicing patterns associated with fusion events. Using various existing tools combined with this custom program, we developed a pipeline for the identification of fusion transcripts applicable as targets for immunotherapy. In addition to identifying candidate neoantigens associated with fusions, we were able to use the pipeline to establish a method for measuring the frequency of fusion events, which correlated to patient outcome, as well as highlight some similarities between canine and human osteosarcomas. The results of this study of osteosarcomas underscores the numerous benefits associated with conducting a thorough analysis of fusion events within cancer samples.

## Introduction

There are numerous fusion-finding algorithms (FusionHunter, FusionMap, FusionFinder MapSplice, deFuse, Bellerophontes, ChimeraScan, and TopHat fusion), which were compared in a number of ways^1,2^. Not only do these fusion detection tools provide very different results, they do not provide the next logical level of analysis, which is predicting the protein changes resulting from the fusion events. The ability to construct the novel proteins generated by fusions provides an unexplored source of somatic mutations that contribute to the neoantigenome.

Somatic mutations in the tumour genome can cause tumours to express neoantigens. These tumour-specific mutant proteins can be processed into short peptides (epitopes) and presented on the surface of tumour cells in the context of major histocompatibility complex (MHC), human leukocyte antigen (HLA) in humans, leading to their immune recognition by T-cells as foreign antigens. Tumour neoantigens can be highly immunogenic because they are not present in normal tissues and thus bypass central thymic tolerance.

Extensive research has indicated that recognition of the tumour neoantigens by the immune system has clinical relevance. Several studies demonstrated a correlation between predicted neoantigen load and both intratumoural immune infiltrate and patient survival^3–7^. Neoantigen-specific T cells have been identified in several human cancers^8–11^. Several studies showed a correlation between predicted neoantigen load and clinical response to checkpoint blockade therapy^12–17^, and that it was the frequency of the neoantigen-specific T cells that increased in the responding patients after therapy^9,13^.

Neoantigens are not only important targets of checkpoint blockade therapy, but they can also be used to develop personalized cancer-specific vaccines. Mouse models^18,19^ and clinical studies^20–22^ have shown robust anti-tumour T-cell responses by using neoantigen-based vaccines. Altogether, these data indicate that neoantigens are ideal tumour rejection antigens and thus, the identification of mutations that can be a source of neoantigens is critical for successful immunotherapy.

To date, most research has focused on identifying neoantigens generated from missense mutations. Gene fusions, especially out-of-frame gene fusions, are an attractive potential source of tumour neoantigens, because, after translation of the first open reading frame, a second novel out-of-frame sequence is translated until a premature stop codon is encountered, thereby encoding long stretches of novel peptides that may contain multiple potential immunogenic epitopes.

To find such neoantigens, one must seek specific types of fusions, such as fusions generated by the joining of chromosomal breaks occurring within introns of both genes involved in the fusion. Most often this will result in a transcript that retains normal splicing patterns with the latter part of the transcript being out-of-frame. However, interesting splicing variations can occur if one of the breaks occurs in a location other than an intron, or if one of the genes is normally transcribed in an orientation opposite to the other gene.

Osteosarcomas (OS) are characterized in human samples by highly disrupted genomes^23^. Furthermore, studies showed half of the juvenile OS analysed displayed the 5 factors characterized by kataegis, a localized pattern of hypermutation, which colocalizes with structural variation breakpoints^24,25^. Rearranged genomes have the potential to generate fused transcripts (fusions) containing components of 2 or more genes. Although fusions can be detected at the genomic level though many methods (i.e. chromosome banding, FISH, PCR), RNA-sequencing (RNA-seq) offers the ability to specifically interrogate the transcribed fusions, looking for unusual splicing patterns, and elements associated with protein generation. Other methods, especially those geared toward evaluating DNA directly, cannot rule out fusions subject to nonsense mediated decay, nor are they able to detect transcription-induced chimeras (TICs).

While evaluating various tools to predict CD8 T-cell neoeptitopes within the novel protein sequences generated by the fusions present in human OS, we found an interesting relationship between CD8 T-cell levels and neoepitope load. Also, we identified an interesting recurring TIC (*TMEM165*-*CLOCK*). We will describe in detail how we analysed this fusion and discuss its implications in metastasis.

Our primary goal for this project was to introduce a bioinformatic blueprint to identify potential tumour-specific neoantigens generated by gene fusions. During this effort we were also able to develop auxiliary methods to measure the frequency of fusion transcripts derived from genomic rearrangements within a sample and correlate it to patient survival in osteosarcomas, identify some similarities between human and canine osteosarcoma fusions and recognize transcripts incapable of producing normal proteins even though they are present at normal levels.

## Results

### Development of an integrated pipeline for identifying novel protein coding sequences

**Figure 1:**
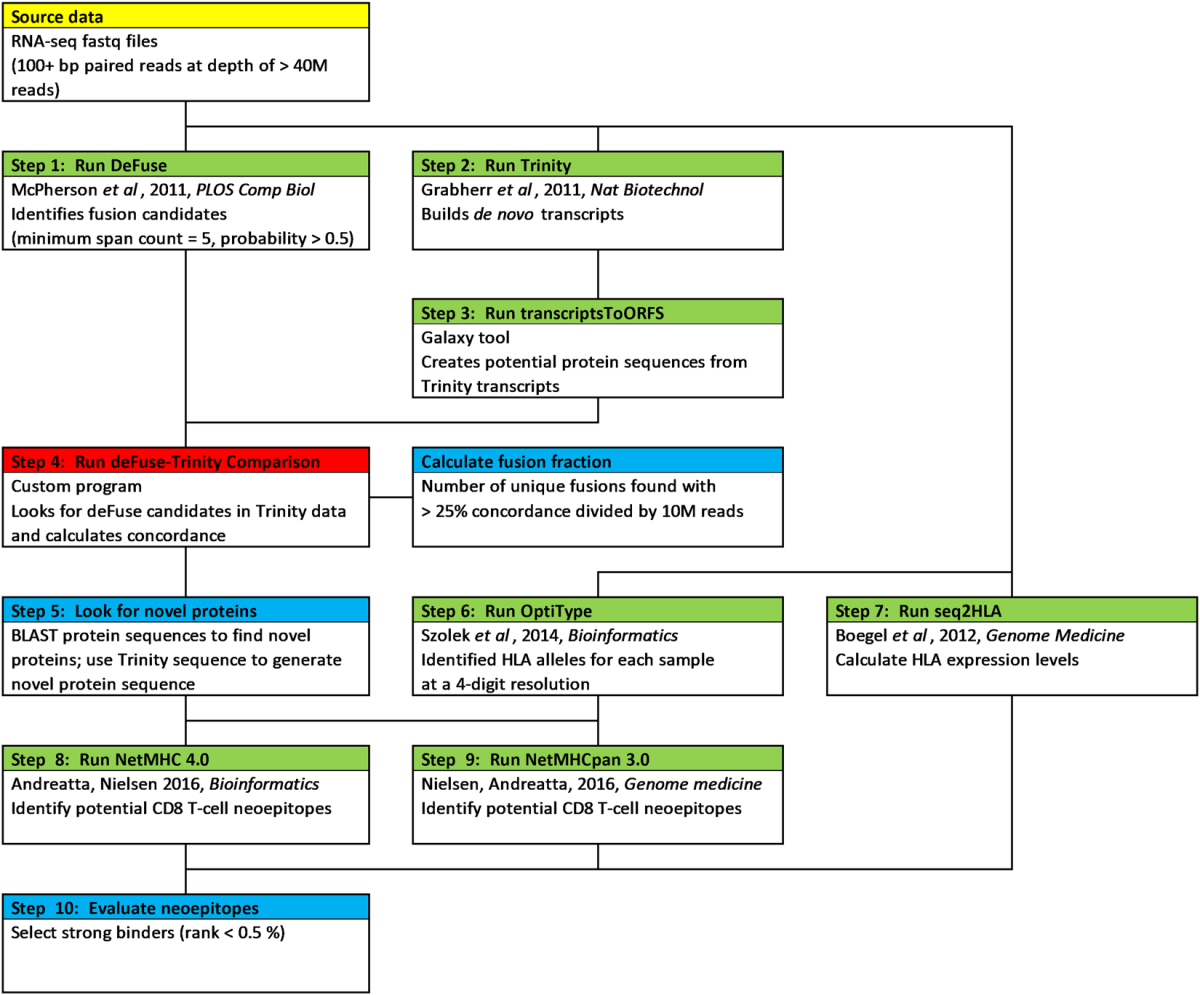
Diagram of the methodology used to identify potential neoantigens in OS. Starting data highlighted in yellow, pre-existing programs highlighted in green, custom programs highlighted in red, and manual processes highlighted in blue.

We used deFuse^26^ to identify all the fusion candidates in various mRNA-seq OS data sets: 12 samples provided by St. Jude Children’s Research Hospital^24^, 35 previously published samples^27^, 32 canine samples collected at the University of Minnesota^28^, 3 normal human osteoblasts^29^, and 1 normal canine osteoblast^28^. We then ran Trinity^30^ to generate *de novo* transcripts independent of a reference genome, and processed the assembled transcripts from Trinity through the transcriptsToORFs program (a Galaxy^31–33^ tool) to predict potential protein products. The outputs from deFuse, Trinity, and transcriptsToORFs were compared by the deFuse-Trinity Comparison program to validate the deFuse candidates. A complete diagram of the pipeline is presented in Fig. 1 with the details of the methodology (Fig. 1, Steps 1–4) described in Supplementary Information.

**Figure 2:**
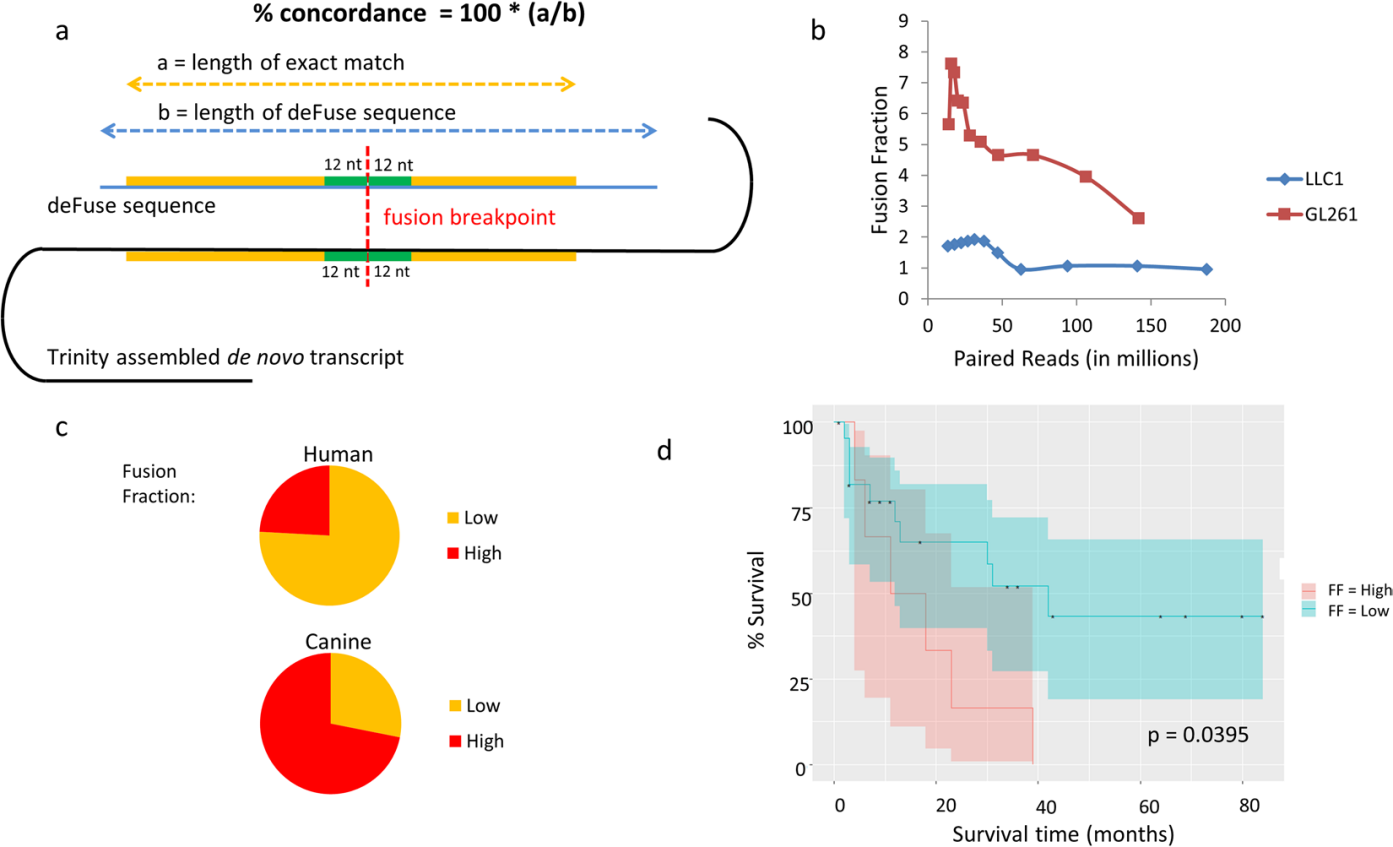
Fusion fraction measurement. (**a**) Diagram depicting the methodology for calculating the fusion fraction. (**b**) Graph showing the fusion fraction relative to selected read depths in mouse cancer samples. (**c**) Graph depicting the number of samples with 2-fold or greater fusion fractions in human and canine samples when compared to the fusion fraction in normal osteoblasts. (**d**) Kaplan-Meier curve showing the survival of patients in the Perry *et al*. data set with a high fusion fraction (FF) of >2.7.

The total number of fusions identified by deFuse, along with the number of fusions verified by Trinity, are listed in Supplementary Table S1. This preliminary tally identified 5 samples of insufficient quality for fusion analysis and they were eliminated from further study. Because the St. Jude cohort had a mixture of primary and metastatic samples, it was then used to illustrate the analytical process. The total number of fusions found by deFuse is shown in Table 1, as well as the number verified by Trinity, with a breakdown by type. The full names of the St. Jude cohort are in Supplementary Table S2. The quality of the samples from St. Jude was excellent with the measure of aligned reads >89.8% for all samples, and the measure of concordant reads >81.3% (Supplementary Table S3). If the fusion identified by deFuse in an OS sample was not also identified in the osteoblast controls, and deFuse and Trinity strongly agreed on the 200–500 nts surrounding the fusion breakpoint (concordance >25%, Fig. 2a), then the Trinity sequence for both the transcript and protein in each of the INTER and INTRA fusions (as defined in Supplementary Information) were BLASTed, and the results analysed to: 1) make sure the sequences around the fusion point were unique to the genes identified, 2) determine the direction of the transcript by analysing the splicing patterns, 3) ensure the beginning of the fusion transcript was located within an open reading frame, and 4) characterize the change to the protein involved, such as whether the fusion was in-frame, contained unusual splicing patterns, or generated novel protein sequences at the beginning or end of the protein (Supplementary Tables S4 and S5). At least 1 putative abnormal protein was detected in each of 12 OS samples, with a maximum of 21 and a median of 3.5 (Table 1). Total number of INTER and INTRA fusions for additional samples are included in Supplementary Table S1.

**Table 1:** Number of fusion candidates identified in the St. Jude cohort of 12 juvenile OS samples.

Sample	Total fusions					Abnormal proteins			
	deFuse	Trinity	INTER	INTRA	TIC	INTER	INTRA	Total	
OS03	66	19	3	4	12	1	0	1	
OS04	69	21	2	1	18	1	0	1	
OS05	117	54	11	6	37	7	2	9	
OS07	128	38	9	5	24	1	2	3	
OS08	68	20	9	0	11	4	0	4	
OS09	394	130	36	22	72	11	10	21	
OS11	52	21	7	5	9	6	4	10	
OS12	143	57	13	8	36	5	1	6	
OS13	45	11	2	3	6	0	1	1	
OS16	35	14	1	2	11	1	1	2	
OS20	175	29	6	3	20	1	0	1	
OS29	37	11	2	2	7	2	2	4	
						40	23	63	Total
								3.5	Median

### The RNA-seq fusion fraction as a measure of genomic instability

To determine the optimal read depth for detecting fusions, deFuse was run on two deeply sequenced mouse RNA-seq samples (LLC1 and GL261), which were sequenced at depths of 188 million and 142 million paired-end reads, respectively. Subsets of the reads were selected from each set of samples, by selecting every nth record (1–10) from each of the paired fastq files, and including a subset composed of every even number record and every other odd numbered record. The RNA-seq fusion fraction was determined by first identifying the number of INTER and INTRA fusions from the deFuse list of fusions with a probability of more than 0.5 (as defined by deFuse) and having a matching junction in the Trinity data containing at least a 25% concordance. The 25% concordance limit was established to eliminate false positives after BLASTing all of the deFuse and Trinity generated sequences. We also excluded *SRGAP2* fusions, which were generated by the incomplete annotation of *SRGAP2* and its duplicate genes (*SRGAP2B*, *SRGAP2C*, and *SRGAP2D*)^34^. By comparing this total number of fusions associated with chromosomal breaks to the total number of mapped reads in the sample (deFuse span count/10 million paired reads), we get a sense of the genomic instability. The evaluation of the LLC1 and GL261 samples demonstrates the fusion fraction is relatively stable at read depths between 40 M and 120 M paired reads, and within this range the number of fusions found in a sample is proportional to the read depth (Fig. 2b).

The fusion fraction was next calculated for the human and canine OS samples and osteoblast controls (Supplementary Table S1). In general, the canine osteoblast and OS samples had higher RNA-seq fusion fractions than human OS. This is probably due to incomplete annotation of isoforms within in the canine genome. Within both the human and canine samples there were samples with high fusion fractions, greater than 2 times the fusion fraction of the normal osteoblasts (Fig. 2c). A survival plot was created for the Perry *et al*. data comparing the samples with high fusion fractions (>2.7), representing the top quartile, with the lower fusion fractions (<2.7). It showed reduced survival in patients with a higher fusion fraction (Fig. 2d).

In addition to calculating the fusion fraction, the entire protein prediction process was conducted on the GL261B and LLC1 samples (Supplementary Table S6). There were 7 total fusions detected. Interestingly, two of the fusions in the GL261 sample were present in the same Trinity transcript, indicating a triple gene fusion made from elements of Tmem2, Rfx3, and Tmc1. All 7 fusions were confirmed by PCR and Sanger sequencing (Supplementary Information).

### TMEM165-CLOCK identified as a recurrent TIC in human OS samples

TICs are transcripts containing components from two or more neighbouring genes and are not necessarily caused by genomic abnormalities, but rather are more likely generated by read through transcription^35^. TICs were found to be more prevalent in prostate cancer cells when compared to normal matching tissue^36^, indicating they may be a cause or consequence of tumour development. The mechanisms driving TICs still need to be elucidated; however, one article described the mechanism behind the creation of the *TSNAX-DISC1* TIC in human endometrial carcinoma cells, which involved a long segment of non-coding RNA between the genes causing interference with the binding of the CTCF protein to insulators^37^. Also, a later study suggested environmental factors, such as serum components or androgens, were key in regulating the binding of CTCF to insulators, which in turn is inversely related to the levels of the *SLC45A3-ELK4* TIC^38^.

Some TICs may be strong vaccine targets and potentially druggable targets if involved in oncogenesis. In addition, they provide some interesting insight into the nature of the cancer cells being studied. We looked for commonly occurring TICs identified by deFuse in 3 or more of the 12 OS samples from the St. Jude cohort and verified by Trinity in 2 or more of the samples (Supplementary Table S7). We used the same methods as described for INTER and INTRA fusions to characterize the TICs. Of the 19 TICs analysed 7 appeared to generate proteins not previously described.

**Figure 3:**
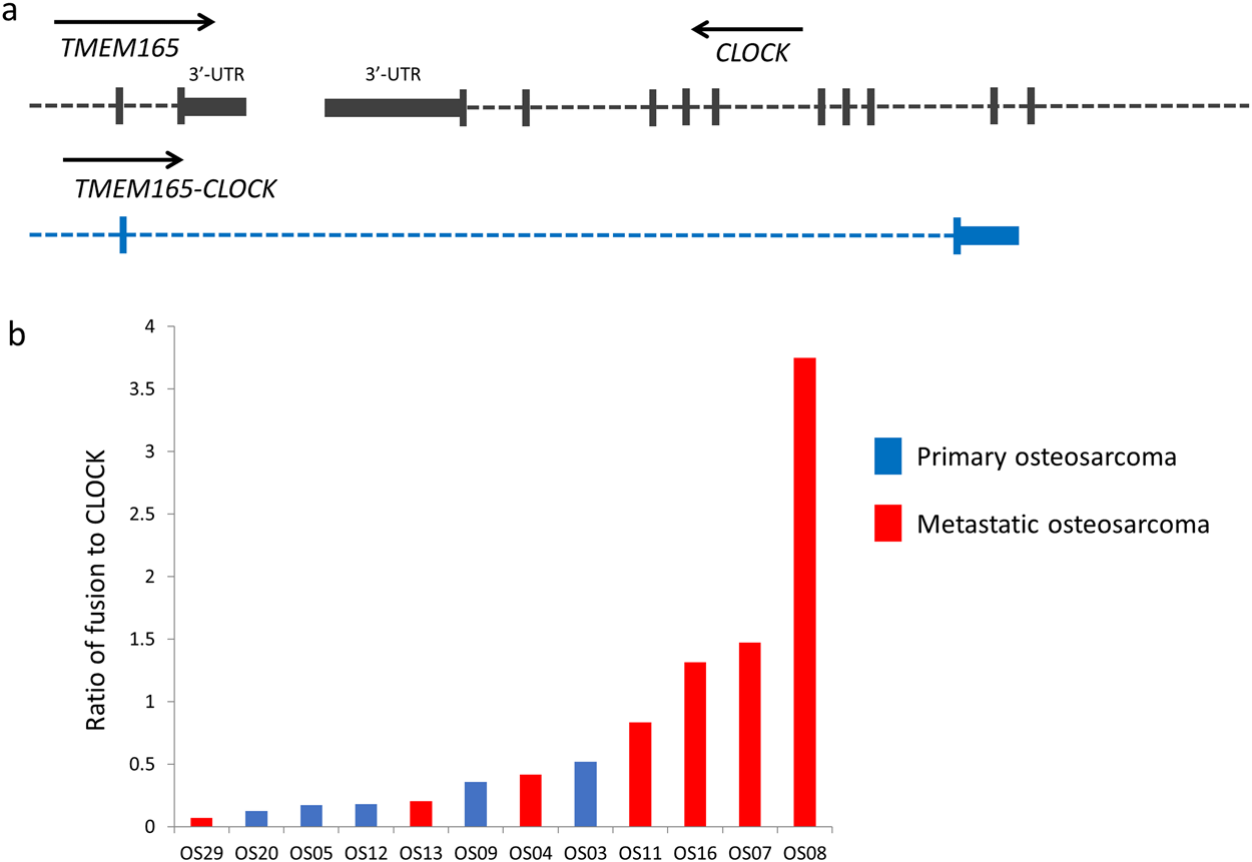
Prevalence of the TMEM165-CLOCK fusion/TIC in metastatic samples. (**a**) Schematic of the TMEM165-CLOCK TIC. (**b**) Ratio of the TMEM165-CLOCK fusion/TIC reads to CLOCK reads in the St. Jude cohort.

One of the TICs identified by this process was *TMEM165*-*CLOCK*, a fusion detected by deFuse in 8 of the 12 OS samples. The *TMEM165* and *CLOCK* genes are adjacent to each other on chromosome 4 but are transcribed in opposite directions (Fig. 3a). Ensembl does contain documentation of a *TMEM165* isoform (013) spanning the fusion location identified by deFuse, however, the transcript appears to be incomplete (missing the first 2 exons from TMEM165).

Trinity predicts the transcript will generate a protein identical to TMEM165 except for the last 27 amino acids (aa). Interestingly, exon 15 of *CLOCK*, which contains 72 nts and is transcribed in both directions, could be a naturally occurring example of antisense RNA capable of blocking translation^39^; specifically, the 3′-UTR of the *TMEM165*-*CLOCK* mRNA may bind to the middle of *CLOCK* transcripts and prevent translation of CLOCK.

If the *TMEM165*-*CLOCK* chimera is serving as an antisense RNA, the abundance of *TMEM165*-*CLOCK* to normal *CLOCK* transcript would be of interest. We quantified the average number of reads in the last 10 nts of exon 15 of *CLOCK*, representing the total number of both *CLOCK* and *TMEM165*-*CLOCK* transcripts, and the first 10 nts of intron 15, representing just the *TMEM165*-*CLOCK* transcripts (Supplementary Table S8). A ratio of the *TMEM165*-*CLOCK* to *CLOCK* was unusually high in most of the metastatic samples from the St. Jude cohort, and although not all the metastatic samples had a high ratio, all the samples with high ratios were metastatic (Fig. 3b).

### Some genes are recurrently altered by gene fusions across samples and species of osteosarcomas

The deFuse-Trinity comparison process (Supplementary Table S9) was run on the 77 high-quality human and canine OS samples listed in Supplementary Table S1. Although there were no fusions identified between 2 genes with identical junction locations, there were 10 pairs of fusions sharing the same 5′ gene, 4 of which had the same breakpoint location in the 5′ gene. (Table 2). In all cases, the fusions were not detected in normal osteoblast samples, making it unlikely the fusions were the result of an annotation problem. Among the 10 common starting genes, 2 are well known tumour suppressor genes (*TP53* and *RB1*), and 3 are associated with osteoblast differentiation (*KAT6A*, *ROCK1*, and *RUNX2*). Disruption of normal expression in the tumour suppressor genes, *TP53* and *RB1*, could contribute to the selection process of the OS cells. *TP53* and *RB1* were already shown to be frequent fusion partners in OS^40,41^, and *Rb1* was shown to be a common insertion site in a Sleeping Beauty transposon mutagenesis screen used to generate OS in mice^29^. However, the role of the fusion modified osteoblast differentiation genes is unclear. *KAT6A* is believed to be a transcriptional coactivator of *RUNX2*^42^, inhibition of *ROCK1* increases osteoblast differentiation^43^, and *RUNX2* is essential for osteoblast differentiation^44^. Furthermore, RUNX2 and ROCK1 were shown to have oncogenic properties in OS^45,46^. Accompanying the *RUNX2* fusion, there was also a large increase in the expression of normal *RUNX2*, which would support the oncogenic nature of RUNX2. Regardless, all the fusions are predicted to cause a radical change to the resulting protein (Supplementary Table S10) and at a sufficient prevalence (Table 2) to make it highly likely Gene 1 (the 5′ gene) would experience a significant change in function, which in many cases is not reflected in the expression levels of Gene 1. The sequences used to search the raw fastq files for the number of reads are provided in Supplementary Table S10.

**Table 2:** DeFuse-Trinity comparison identified fusions with a common starting gene. Osteoblast expression levels for Gene 1 were obtained from normal human and canine samples^28^.

Sample	Gene 1	Gene 2	Nbr. of reads		Prevalence of fusion	FPKMs		
						Osteo-blast	Osteosarcoma	
			Fusion	Normal			Gene 1	Fold chg.
BZ27	*KAT6A*	*MLLT10P2*	18	14	0.56	4.216	7.129	1.691
OS29	*KAT6A*	*PBK*	7	4	0.64	4.216	5.742	1.362
DOS37	*ARFGEF3*	*CITED2*	8	1	0.89	0.412	5.391	13.074
BZ01	*ARFGEF3*	*Metazoa_SRP*	15	0	1.00	0.100	0.226	2.268
HO010	*KIF16B*	*SORBS2*	10	5	0.67	3.644	3.919	1.076
BZ08	*KIF16B*	*TASP1*	24	6	0.80	3.644	3.424	−1.064
BZ33	*RB1*	*PRKG1*	8	48	0.14	11.796	9.100	−1.296
BZ14	*RB1*	*SDCCAG3P2*	21	26	0.45	11.796	4.737	−2.490
BZ36	*ROCK1*	*7SK*	8	23	0.26	13.705	8.518	−1.609
OS07	*ROCK1*	*VAPA*	38	20	0.66	13.705	6.275	−2.184
BZ38	*RUNX2*	*KLKP1*	13	59	0.18	6.069	56.434	9.298
HO047M	*RUNX2*	*LRRTM4*	21	51	0.29	6.069	75.984	12.520
DOS112	*TMEM67*	*C8orf37*	10	15	0.40	2.784	8.572	3.080
OS09	*TMEM67*	*GDF6*	31	18	0.63	4.117	3.192	−1.290
HO036	*TP53*	*HMGB1P46*	41	10	0.80	26.864	8.159	−3.292
BZ03	*TP53*	*ZPBP*	11	25	0.31	26.864	14.206	−1.891
OS09	*USP25*	*CHODL-AS1*	91	173	0.34	18.964	17.635	−1.075
DOS6M	*USP25*	*SLC36A4*	31	32	0.49	4.566	9.552	2.092
OS09	*VPS13B*	*RP11-1057N3.1*	149	42	0.78	4.667	13.157	2.819
DOS6M	*VPS13B*	*ZZEF1*	5	25	0.17	4.915	7.211	1.467

### Potential strong binding CD8 T-cell neoepitopes derived from fusions identified in all OS patient samples evaluated

**Figure 4:**
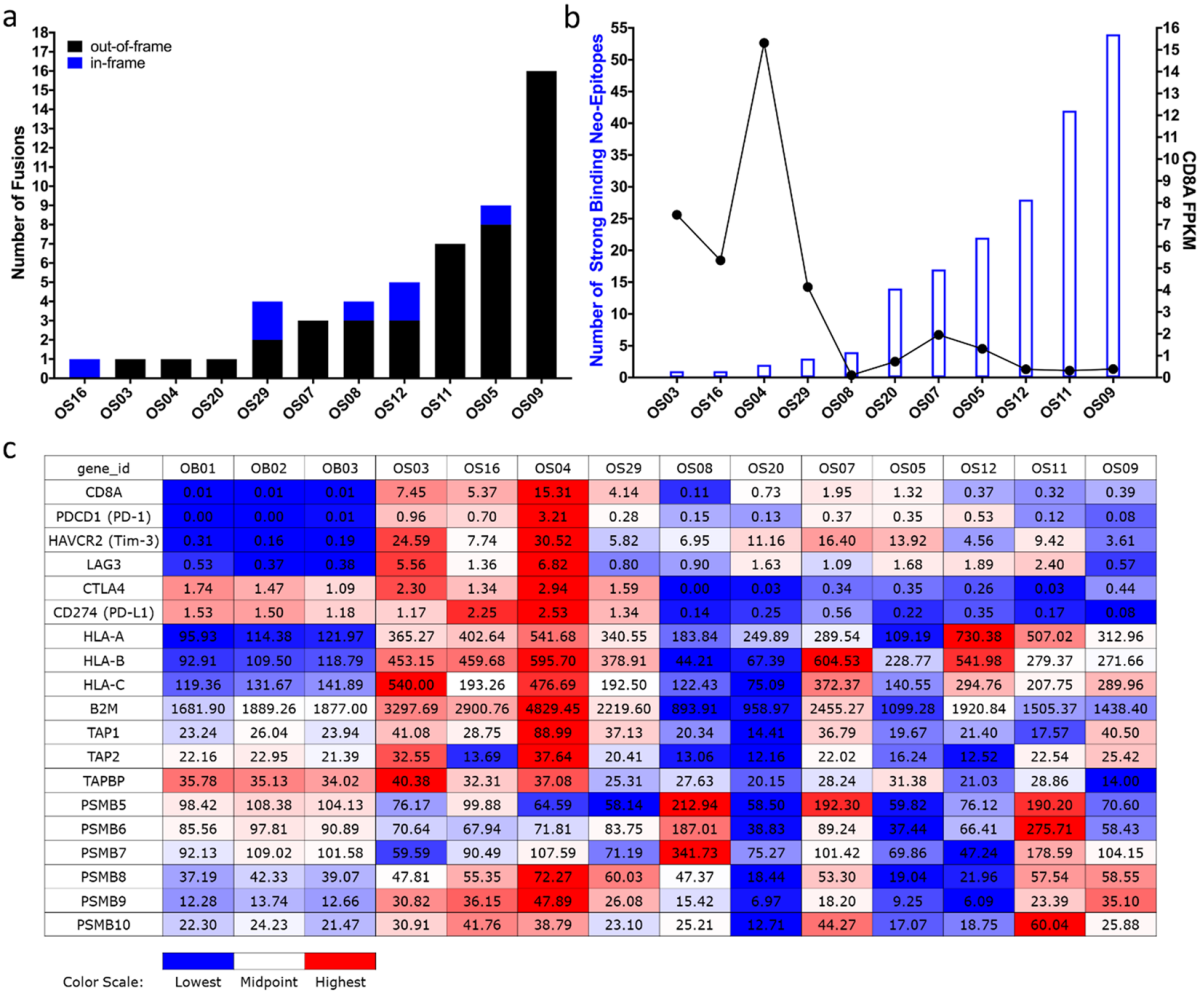
(**a**) Number of fusions identified in OS tumours in the St. Jude cohort, (**b**) Number of predicted CD8 T-cell neoepitopes with strong binding to patient-specific HLAs, and *CD8A* expression (indicator for CD8+ TIL level) in OS tumours in the St. Jude cohort of 11 patients. Statistical analysis indicates a significant strong negative correlation between the two variables (r = −0.5583, r^2^ = 0.3117, p = 0.0371*), (**c**) Gene expression values and levels presented in the same order as graph B for *CD8A*, CD8 T-cell inhibitory receptors: *PD-1*, *Tim-3*, *LAG-3*, *CTLA4*, and inhibitory ligand *PD-L1*, and several antigen processing and presenting machinery components: *HLA class I* (*A, B, C*), *B2M*, *TAP1*, *TAP2*, *TAPBP*, *PSMB5*, *PSMB6*, *PSMB7*, *PSMB8*, *PSMB9*, *PSMB10*. Expression values are reported as FPKM for all genes with the exception of *HLAs* that are reported as RPKM. OS = osteosarcoma tumours, OB = osteoblasts controls.

**Figure 5:**
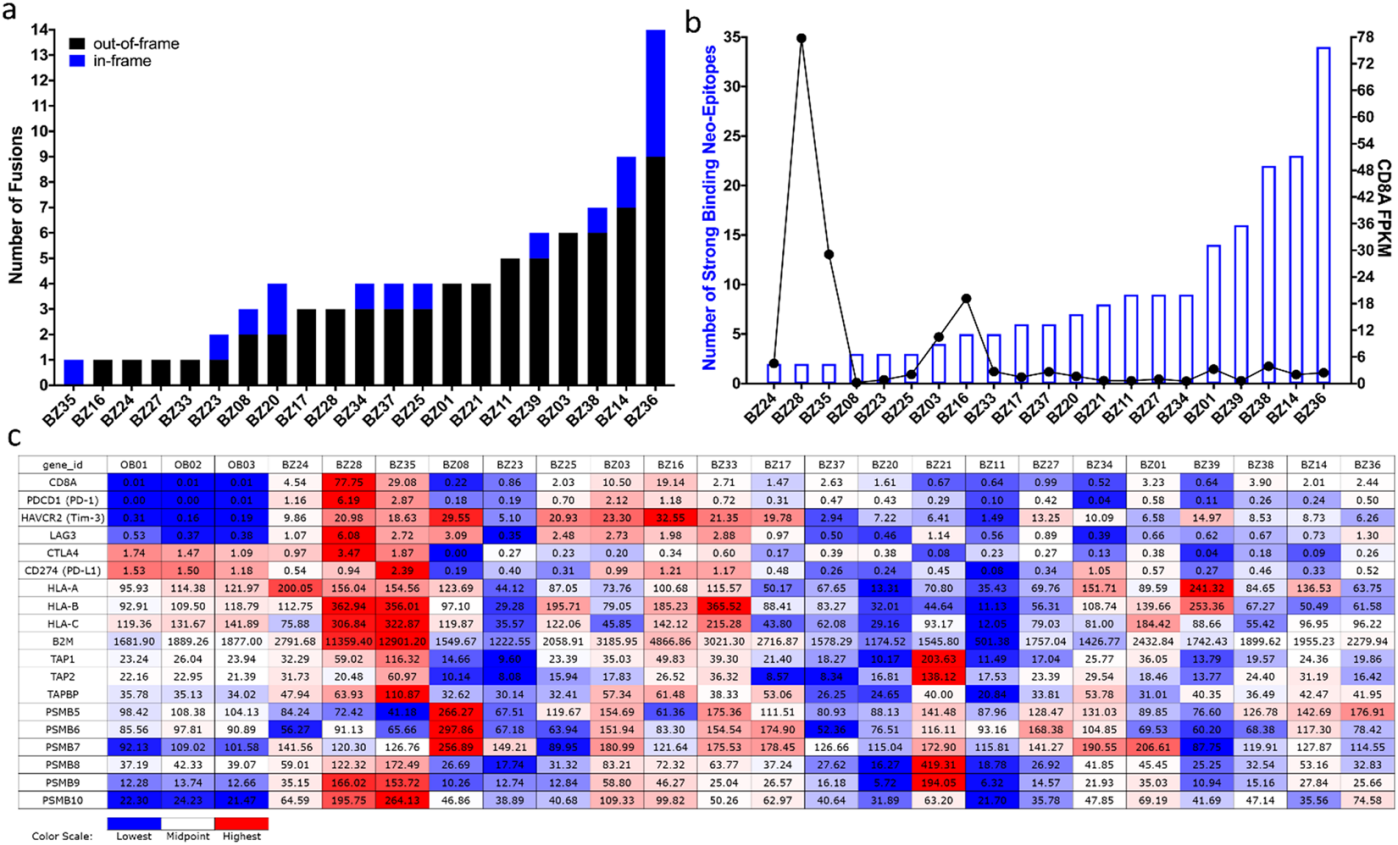
(**a**) Number of fusions identified in osteosarcoma tumours in the Perry *et al*. data set, (**b**) Number of predicted CD8 T-cell neoepitopes with strong binding to patient-specific HLAs, and *CD8A* expression (indicator for CD8+ TIL level) in OS tumours in the Perry *et al*. data set of 21 patients. Statistical analysis indicates a weak/moderate negative correlation between the two variables (r = −0.2798, r^2^ = 0.07826, p = 0.1097), (**c**) Gene expression values and levels presented in the same order as graph B for *CD8A*, CD8 T-cell inhibitory receptors: *PD-1*, *Tim-3*, *LAG-3*, *CTLA4*, and inhibitory ligand *PD-L1*, and several antigen processing and presenting machinery components: *HLA class I* (*A, B, C*), *B2M*, *TAP1*, *TAP2*, *TAPBP*, *PSMB5*, *PSMB6*, *PSMB7*, *PSMB8*, *PSMB9*, *PSMB10*. Expression values are reported as FPKM for all genes with the exception of *HLAs* that are reported as RPKM. BZ = osteosarcoma tumours, OB = osteoblasts controls.

Our analysis of INTER- and INTRA-chromosomal fusions within the OS tumours identified both in-frame and out-of-frame fusions. The out-of-frame fusions outnumbered the in-frame ones (Figs 4a and 5a) providing thus, an enriched source of neoantigens through their longer out-of-frame novel sequences.

We evaluated all the neoantigens generated by the in-frame and out-of-frame fusions for potential CD8 T-cell neoepitopes using NetMHC 4.0^47^ and NetMHCpan 3.0^48^ prediction algorithms. We focused our analysis on neoepitopes (8- to 11-aa in length) that were predicted to have strong (rank <0.5%) binding to patient-specific HLAs (Supplementary Tables S11 and S12). We used the OptiType algorithm^49^ to predict at four-digit resolution the HLA alleles carried by each patient (Supplementary Table S13) using RNA-seq data. We found a wide range of predicted strong binding CD8 T-cell neoepitopes derived from fusions, from 1 to 54 neoepitopes per patient (Figs 4b and 5b). We also evaluated the 22 aa sequence specific to the most prominent TIC, *TMEM165-CLOCK*, and found strong binding epitopes to patient-specific HLAs in 7 out of the 12 St. Jude OS tumours (Supplementary Table S14).

### Predicted neoepitope load from fusions negatively correlates with CD8A expression

To see if there is a relationship between the predicted neoepitope load from fusions and CD8+tumour-infiltrating lymphocyte (TIL) levels, we used *CD8A* gene expression from RNA-seq data^6,28^ as an indicator for CD8+TIL level. All OS tumours had *CD8A* gene expression of varying degrees (Figs 4c and 5c, Supplementary Figs S2a and S4a) suggestive of CD8+ T-cell infiltration. We observed two OS tumour groups that had an inverse relationship between the predicted neoepitope load from fusions and CD8+ TIL levels. One group of tumours, including OS04, OS03 and OS16 (Fig. 4b,c, Supplementary Fig. S2a), and BZ28, BZ35 and BZ16 (Fig. 5b,c, Supplementary Fig. S4a) had low predicted neoepitope load from fusions but high levels of CD8+ TIL. This suggests that in these OS tumours, CD8+ TIL may infiltrate the tumours in response to other immunogenic mutations as well, such as missense mutations. Another group of tumours, including OS09 and OS11 (Fig. 4b,c, Supplementary Fig. S2a), and BZ36, BZ14 and BZ38 (Fig. 5b,c, Supplementary Fig. S4a) had high predicted neoepitope load from fusions but low levels of CD8+ TIL. Possible explanations for the low level of CD8+ TIL despite high predicted neoepitope load might be inefficient T-cell priming, immunodominance of some neoepitopes, deficient T-cell repertoire of patients, or low/lack of neoepitope presentation on the tumour cells due to defects in expression of some antigen processing and presenting machinery (APM) components.

Since previous studies have shown that the level of CD8+ TIL directly correlates with the expression of several APM components in different tumours^50–52^, we decided to investigate the expression level of several APM components (*HLA class I, B2M, TAP1, TAP2, TAPBP*, constitutive proteasome: *PSMB5, PSMB6, PSMB7*, and immunoproteasome: *PSMB8, PSMB9, PSMB10*) in the OS tumours using RNA-seq data. With the exception of OS03, OS04, BZ16 and BZ35 tumours that had the highest *CD8A* gene expression and no downregulation of expression of the analysed APM components, all the other OS tumours had downregulation of expression of the analysed APM components that most likely interfere with neoepitope processing and presentation by tumour cells (Figs 4c and 5c, Supplementary Figs S3 and S5).

Since all OS tumours had CD8+ T-cell infiltration (of varying degrees) inferred from *CD8A* gene expression, we wanted to investigate the expression level of several T-cell inhibitory receptors *PD-1, Tim-3, LAG-3*, and *CTLA4* using the RNA-seq data to assess the functional state of these CD8 + TILs. We found increased expression of *PD-1* and *Tim-3* in all OS tumours and increased expression of *LAG-3* in the majority of tumours, whereas *CTLA4* had increased expression in fewer tumours (Figs 4c and 5c, Supplementary Figs S2b–e and S4b–e). The OS tumours OS04 and OS03 (Fig. 4c, Supplementary Fig. S2a–e), and BZ28 and BZ35 (Fig. 5c, Supplementary Fig. S4a–e) that had the highest *CD8A* gene expression, also had high expression of *PD-1, Tim-3, LAG-3 and CTLA4*, indicative of a complex dysfunctional state of the CD8 + TILs in these tumours.

## Discussion

Expression analysis of RNA-seq data only provides a limited picture of cancer development. Fusion analysis can add an important piece to the cancer puzzle by identifying genes subjected to functional changes, especially in cancers characterized by many genomic rearrangements, such as OS^23^. Furthermore, it can assist in identifying previously undocumented TICs, as well as potential neoantigens.

For the purpose of identifying tumour-specific neoantigens we developed a strategy for validating fusion candidates from deFuse by comparing the deFuse provided fusion sequence to the *de novo* transcriptome generated by Trinity (Fig. 1). This fusion analysis pipeline includes methods to predict neoantigen load from fusion derived frameshifts and to quantify the incidence of fusions in a single sample, which we refer to as the “fusion fraction”. The RNA-seq fusion fraction is useful to quantify the level of genomic instability in a cancer sample, and can be used to separate OS into subgroups, which may be an indicator of prognosis and patient response to current treatment options.

Since the number of fusions, as detected by the deFuse-Trinity comparison program, is dependent on the read depth, an adequate read depth is essential. It is important to be able to locate the fusions, and yet, the span count of each fusion needs to be of sufficient quantity to predict adequate protein to serve as a vaccine target. In addition, since OS tumours are so highly heterogeneous, the fusions with higher levels of expression are more likely to occur in a higher percentage of the tumour cell population. We recommend a minimum of 40 million paired-end reads to find a sufficient number of neoantigens. At lesser depths Trinity is unable to build complete *de novo* transcripts for many of the prominent fusion transcripts, as exemplified by the mouse samples, which were sequenced at large depths and then processed through the fusion analysis pipeline in subsets of varying depths.

Some apparent fusion transcripts and TICs are likely not tumour-specific, and although it is ideal to have matched normal samples to serve as a control to eliminate “false positives” stemming from incomplete or inaccurate genomic mapping, the use of normal cells from similar tissues (even if not from the same patient) can still assist in eliminating many of these so-called fusions from consideration. In the case of the 12 juvenile OS samples, we used 3 human osteoblast samples. To refine this process for a clinical setting, for generating a list of vaccine targets for a specific patient, a normal matching tissue sample would be required. We would also recommend confirmation of the fusion breakpoint in the DNA of the cancer sample.

INTER and INTRA fusions are the best candidates for vaccines targets, since they are not likely to occur in any normal tissues within the patient. However, this does not mean TICs should be ignored. There may be some TICs unique to cancer progression, which may also serve as vaccine targets, or provide druggable targets. In addition, the study of the TICs can provide some interesting information regarding disease progression.

There are several reports of TICs being identified in human tumours, such as *GOLM1-MAK10* in oesophageal squamous cell carcinoma^53^ and *TMEM79-SMG5*, which was suggested as a diagnostic marker in prostate cancer^36^. However, their role in tumorigenesis has not been fully elucidated. We believe the analysis of the *TMEM165*-*CLOCK* TIC in OS will be equally challenging. The *TMEM165-CLOCK* TIC appears to be quite prevalent in most metastatic OS samples studied here. It was also identified as commonly found in prostate cancer^36^. But, what would single cell RNA-seq show? Is the ratio constant across each individual cell? Also, its role in metastasis is unclear. Is the change in the C-terminus of the TMEM165 protein involved in its metastatic role, or does the *TMEM165*-*CLOCK* transcript serve as an antisense inhibitor of normal CLOCK translation? Inhibition of normal TMEM165 in zebra fish affects bone development and osteoblast differentiation^54^. Loss of expression of circadian clock associated genes is associated with more aggressive forms of breast cancer^55^. Unravelling the role of the *TMEM165-CLOCK* TIC in the metastatic process could lead to the identification of druggable targets. Interestingly, the expression levels for the individual genes, *TMEM165* and *CLOCK*, did not vary significantly between the OS and osteoblasts, and thus provides an example of potential oncogenic changes that cannot be detected by expression analysis alone.

As expected, the genomic instability of OS led to numerous fusions within the sets of samples we analysed, and although there were no common fusions found, there were numerous genes appearing at the 5′ end of the fusion in more than one sample (*TP53*, *RB1*, *RUNX2*, *ROCK1*, *KAT6A*, *ARFGEF3*, *KIF16B*, *TMEM67*, *USP25*, and *VPS13B*). In the case of the tumour suppressors, such as *TP53* and *RB1*, the loss of function of the 5′ gene of the fusion is an obvious contributor to tumorigenesis. However, the role the *RUNX2* fusions is baffling. The samples with *RUNX2* fusions experienced a spike in expression of the normal *RUNX2* transcripts. Perhaps this is a result of a loss of the N-terminal autoinhibitory region of *RUNX2*^56^, or it may introduce some other type of functional change to the *RUNX2* complex. Unravelling the role of *RUNX2* fusions will be challenging.

The results of our study have several clinical implications. Using the OS RNA-seq data we identified several gene fusions, and the predicted tumour-specific neoepitopes generated by these fusions could be used to design personalized immunotherapy protocols (vaccines or adoptive T-cell therapies) to induce or enhance anti-tumour immune responses. The high presence of fusions in a subset of canine OS (Fig. 2c) and the common starting genes found between the canine and human fusions demonstrate further similarities in OS between humans and canines, to be added to those previously described^28^ With the much higher incidence of OS in canines, there are compelling reasons to begin testing patient-specific vaccines in dogs prior to human clinical trials. Further, interrogating the tumour transcriptome allows optimization of personalized immunotherapy protocols. Evaluating the tumour immune infiltrate, the CD8+ TILs, and the expression of inhibitory T-cell receptors may indicate the patients who are potential candidates for checkpoint blockade therapy. Also, evaluating potential immune escape mechanisms in tumours, such as defects in expression of MHC class I antigen processing and presenting machinery components, may indicate the patients who would benefit from therapy protocols designed to upregulate neoantigen processing and presentation by tumour cells, thus increasing the level of tumour cell recognition by the immune system.

Here we provide a digital approach to quantify fusions (RNA-seq fusion fraction) and to detect prominent fusions using RNA-seq data, deFuse, Trinity, and a novel deFuse-Trinity comparison program (Fig. 1, steps 1–4), plus strategies for analysing the candidate fusions as part of a personalized immunotherapy protocol (Fig. 1, steps 5–10). Using these methods for studying OS we found patient-specific fusions, as well as common TICs detected in all the patient OS samples evaluated. We also identified likely neoantigens generated in the cancer cells, as the result of the fusions, which could potentially serve as patient-specific vaccine targets.

Although our bioinformatic pipeline for fusion analysis predicts neoepitopes without functional validation, it does greatly reduce the candidate fusions and neoepitopes to be considered. For example, in the 30 Perry *et al*. samples evaluated, deFuse identified 4695 fusions, 1094 of which were unique. Using the deFuse-Trinity comparison program, 197 (or 18%) of the unique fusions were found to have a >25% concordance and were predicted to be protein coding. Subsequent BLASTing of the proteins identified 53 with novel protein sequences, which were then fed into the NetMHC algorithms for epitope prediction.

In addition to identifying candidate tumour-specific neoantigens and predicted neoepitopes, we clearly show how expression analysis can be distorted by the presence of fusions in cancer cells, thereby demonstrating the importance of conducting fusion analysis together with expression studies. We were also able to glean some valuable information from a prominent TIC (*TMEM165*-*CLOCK*), which may provide insights into the transformation of primary cancers to metastatic cells. It is conceivable the use of fusion analysis tools will assist in identifying other such genetic anomalies. We hope these techniques will assist our fellow researchers and clinicians in their analysis of RNA-seq identified fusions in a wide variety of cancer types.

## Materials and Methods

### Cell lines

LLC1 mouse Lewis Lung Carcinoma cells were acquired from ATCC (Manassas, VA), item CRL-1642. GL261 mouse glioma cells were a gift from the John Olhfest lab. Both cell lines were grown in DMEM/High Glucose (Dulbecco’s High Glucose Modified Eagles Medium), 10%FBS (Fetal Bovine Serum) and 1x Penicillin Streptomycin (Cellgro) on tissue-culture treated plates at 37 °C with 5% CO_2_.

### RNA-sequencing

RNA isolations for the LLC1 and GL261 samples were performed using the RNeasy® Midi Kit (QIAGEN, Venlo, Netherlands). Sequencing was accomplished on the HiSeq. 2000 (Illumina Inc., San Diego, CA) and generated a minimum of 140 million paired-end reads with a length of 50 nts. The resulting RNA-seq data, as well as publicly available human RNA-seq data, were mapped using TopHat2^57^ v2.0.9 against the mouse (mm10) and the human (hg19) genomes, respectively. Mapped data were visualized using Integrative Genomic Viewer (IGV)^58^.

### DeFuse-Trinity comparison

DeFuse (Galaxy^31–33^ Tool Version 1.6.1), Trinity and transcriptsToOrfs (Galaxy Tool Version 0.0.2) were executed on the raw fastq data generated from the RNA-seq. We developed a new application, defuse_trinity_analysis.py, to compare the deFuse identified gene fusions with Trinity transcripts produced by *de novo* assembly of the RNA-seq data. A detailed description of the deFuse-Trinity comparison program is provided in Supplementary Information. The survival plot was generated using ggplot2^59^. The p-value was calculated using survdiff. Both tools were downloaded from CRAN (https://CRAN) and used in R studio 2016 (http://www.rstudio.com/). The defuse_trinity_analysis.py application can be found in the Galaxy toolshed: https://toolshed.g2.bx.psu.edu/view/jjohnson/defuse/b22f8634ff84.

### Verification of fusions

PCR primers specific to the putative fusions were designed using Primer 3^60^ and verified for specificity using Primer-Blast^61^. Standard PCR reactions were performed using cDNA synthesized by Transcriptor First Strand cDNA Synthesis Kit (Roche) using both random hexamer primers and anchored-oligo(dT)_18_ primers. PCR amplicons were confirmed by size on a 1% agarose gel, gel extracted and purified (QIAquick Gel Extraction Kit, Qiagen), and the sequence confirmed by Sanger sequencing.

### Prediction of HLA-binding peptides from in-frame and out-of-frame fusions

We evaluated all the neoantigens generated by the in-frame and out-of-frame fusions for potential CD8 T-cell neoepitopes using NetMHC 4.0 and NetMHCpan 3.0 prediction algorithms. For the in-frame fusions, the protein region considered for neoepitope prediction comprised 11 aa upstream and 11 aa downstream of the fusion point. For the out-of-frame fusions, the protein region considered for neoepitope prediction comprised 11 aa upstream of the fusion point and the entire novel protein sequence from the fusion point until the first downstream premature stop codon. We focused our analysis on neoepitopes (8 to 11 aa in length) that were predicted to have strong (rank <0.5%) binding to patient-specific HLAs. We used the OptiType algorithm to predict, using RNA-seq data, at four-digit resolution, the HLA alleles of each patient. We used seq. 2HLA algorithm^62^ to determine the HLA expression from RNA-seq data.

## Electronic supplementary material

Supplementary Information

Dataset 1

## Data Availability

RNA-seq data for the LLC1 and GL261 samples are available online in the Gene Expression Omnibus at http://www.ncbi.nlm.nih.gov/geo/ (accession number GSE97975).
