# Supplementary material for: Identification of candidate neoantigens produced by fusion transcripts in human osteosarcomas
Source: Sci Rep. 2019 Jan 23;9:358. doi: 10.1038/s41598-018-36840-z (PMC6344567; doi:10.1038/s41598-018-36840-z)
Supplement: Supplementary file 1 — Supplementary Information [file 41598_2018_36840_MOESM1_ESM.docx]

**Supplementary Information**

**Identification of candidate neoantigens produced by fusion transcripts in human osteosarcomas**

**Susan K. Rathe^1*^, Flavia E. Popescu^1^, James E. Johnson^2^, Adrienne L. Watson^1^, Tracy A. Marko^1^, Branden S. Moriarity^1,4,5^, John R. Ohlfest^5^, David A. Largaespada^1,3,4,5^**

^1^Masonic Cancer Center, University of Minnesota, Minneapolis, MN, USA

^2^Supercomputing Institute, University of Minnesota, Minneapolis, MN, USA

^3^Department of Genetics, Cell Biology and Development, University of Minnesota, Minneapolis, MN, USA

^4^Center for Genome Engineering, University of Minnesota, Minneapolis, MN, USA

^5^Department of Pediatrics, University of Minnesota, Minneapolis, MN, USA

*Corresponding author:

Susan K. Rathe

rath0096@umn.edu

***Description of the analytical approach to detect gene fusions***

To understand how gene fusions are contributing to both primary OS and metastases, and to identify potential vaccine and druggable targets, we developed a comprehensive approach to study fusions using RNA-seq data. Although many of the various fusion identification algorithms could be used with this approach, we selected deFuse^1^ because it was already incorporated into the Galaxy tool kit^2-4^, it was thoroughly tested using ovarian and sarcoma samples^1^, it has a very low false-discovery rate when identifying TICs^5^, it was shown to be more sensitive when compared to other tools using just RNA-seq data^6^, and it provides, in its output, an RNA sequence of sufficient length to be used for comparison to the Trinity^7^ output.

However, deFuse only provides a total fusion sequence of 200-500 nucleotides (nts) around the fusion breakpoint. This is insufficient to predict the effect of the fusion on protein production. To get a view of the full transcript containing the fusion, we used Trinity to build *de novo* transcripts from the RNA-seq data. We then created a defuse-Trinity comparison program to take a predefined number of nts on either side of the fusion breakpoint identified by deFuse, and search for the same sequence within the assembled transcripts from Trinity, using both the forward and reverse complement sequences, to determine if Trinity detected the same fusion breakpoint. We used transcriptToOrfs output to see if Trinity predicted the possible generation of a protein from the projected fusion transcript. We elected to use 12 nts on either side of the fusion breakpoint for the Trinity search. In our experience the use of 24 nts for matching minimized the probability of matching to an incorrect transcript. Subsequent Ensembl^8^ BLAST searches helped to identify whether the resulting proteins were in-frame or out-of-frame.

The fusion candidates from this analysis are divided into 3 groups: inter-chromosomal (INTER) fusions where the fused genes are normally located on different chromosomes, intra-chromosomal (INTRA) fusions where the fused genes are normally found greater than 1 million nts apart on the same chromosome, and TICs, which are identified as fusions, but are actually between adjacent genes less than 1 million nts apart and are probably not caused by chromosomal rearrangements. The selection of 1 million nts to distinguish between INTRA fusions and TICs is modifiable in the deFuse-Trinity comparison program and should not be considered absolute in defining the type of fusion. There may be fusions appearing to be TICs, which are caused by genomic breaks, as well as fusions characterized as INTRA, but are most likely TICs.

DeFuse identifies the general location of the fusion breakpoint by specifying “coding”, “intron”, “utr3p”, “utr5p”, “upstream”, or “downstream” for the 2 genes involved. If the breakpoint for one of the genes is within a coding region, it is more likely to produce a protein than fusions involved in upstream or downstream locations. In the final compiled group of 63 INTER and INTRA fusions projected to generate abnormal proteins in the St. Jude cohort, all of the fusions identified were unique to a single OS sample. 53 of the fusions were designated by deFuse as either going from or to a coding region, the most logical location for protein producing fusions. Of the remaining 10 fusions, 9 were going to or from an intron, indicating unusual splicing patterns resulting from the fusion, or the presence of undocumented isoforms. The 10^th^ was defined by deFuse as going from a downstream area of one gene to a 5’-UTR of the other gene, however this was not a correct characterization by deFuse, as this went into the coding region of one of the known isoforms.

We found, in our analysis of the deFuse candidates, the ones projected to generate novel protein sequences started or ended in a coding region or intron. Also, as expected, fusions frequently go from a splice donor site of one gene to a splice acceptor site of the next gene. This indicates the fusion at the DNA level occurred within introns of both genes and the splicing mechanisms properly spliced out the affected introns. Furthermore, through our manual analysis efforts, we concluded the presence of fusions in normal tissues is primarily due to incomplete or inaccurate genomic sequencing, or the mapping of reads to multiple locations within the genome.

***Description of deFuse-Trinity comparison program***

The deFuse application identifies gene fusions from RNA-seq data by analysing discordant paired alignments.   The results include the sequence surrounding the identified fusion boundary. The Trinity application performs *de novo* assembly of RNA-seq reads to reconstruct transcripts. The transcriptsToOrfs tool converts the transcripts to potential peptide sequences. The defuse_trinity_analysis.py application compares deFuse fusion predictions with the *de novo* assembled transcripts produced by Trinity.

First, the application parses the deFuse results, determining the RNA sequence across the breakpoint and characterizing the fusion as: INTER, INTRA, or TIC.  The classification as a TIC depends on a user supplied parameter for the maximum distance in base pairs.

Next a subsequence across the fusion breakpoint is selected, based on the user parameter specifying the number of bases. Then each subsequence and its reverse complement is compared the Trinity transcripts.  Each transcript that contains the subsequence is reported along with the percentage of the full deFuse sequence that was matched by the Trinity transcript, referred to as percent concordance.

Finally, the application reports the Trinity ORFs for matching Trinity transcripts. The ORFs can be extended to include the amino acids following a specified number of stop codons or until the end of the assembled transcript is reached.  The nucleotide sequence of the stop codons is also reported, since the incidence of translational read through varies depending on the specific codon.

***Sanger Sequencing Results of LLC1 and GL261 Fusions***

The ab1 files from the Sanger sequencing were viewed to determine the high-quality sequences and the questionable sequences. The high-quality sequences were BLASTed to determine the location in the mouse genome. Primer sequences available in **Supplementary Table S6**.

LLC1 fusion: Upf2-Rsu1

GGGAGAGGTGAGAGAGGGAGGGGGGGAGTGACGGGATAATGGAGAGAAACAATCAGATGAGGTGATAGCAGATGGTGGACGTGTTAGTTCGGAGGCAAAGAAAGCAAGCGGCATCGGGTAATGGTCCTCTCGCAGGTGATTAAGGAGGCAGGAGGGAGAGGGGAGGGGTGGGCATGTGAGCTGGAAGTGATGATGGTTAAGGGGGAAGGGATAAAGTGAGTGCAAAGGGGATTTTTGAAAAAGATGCTGTCGCCGGAGTTCTTATTGGAAGGGGTTTATGGCATTATAATGACTGCAGTCATTTTGAGGAGCAATGCAGGTTTTCTGTTATGGAGCATGAGACACACTGTCCTATCCGAGAAAATGAAAATTTTCTCCCCAATCC//TGAGGACCAGTGTGCTTTTGCCTCTACGGCAGACACATGCAAGCGAACCCAGAACCTCCAAAGAAGAATAACGACAAATCAAAAAAGATCAGCCGGAAACCC//CTAGCAGCCAGACACTGCG

1-19 (2:6018999-6019017, forward, Upf2, end of exon 11) 100.00% match

20-102 (reverse, 2:13077526-13077608, intron 8 Rsu1) 100.00% match

LLC1 fusion: Slc39a9-Tuba3b

GGTCAGTGTCGATTGGCAGCTCC//CATGTGCATTCCAGTGATGTTTGTGATGTCTGTGGCCAGATTGTCCTTCAGTTCTGGGAAAAGCCTCCGGGTCAAAGATGCAGTGACTGGGAGCTGGCAGAGGCCCAAGAACATGAAAACGCCAAGGAGTGAAGAAA//CAATGGACATCGCGCTCTCCACTTCGGAATAAAGGTTGTTTTCTGGGGGGGGGGGGGGGGGGGTTGGCGGGCCACATAAACCTTAACGGTAACAAACCCGGGGGTCATTCTCTATTTT

1-18 (forward, 12:80666717-80666734, Slc39a9, end of exon 3) 100.00% match

19-136 (reverse, 6:145638440-145638557, upstream of Tuba3b) 100.00% match

GL261 fusion: Micu1-Tmem5

CGTTAGCTGC//ATGAGCACTGTGTAGGACTGGACAATGCAGTTCTGGAGAGGACTGTTGTGAGGAAGATGTAGTCAGAGAAGGAGATGAGCCCACACTCTCCAAGGCTGTAGAAGATGCTGCCTTCATCAGCAAATTTTTCTCGTTCCTGGGCAATTTT//CAGAGTGGTGAGGATGATGTCGTAGAAGGAGGAGAGCACCCCCCACCCTCCGCTGTAGAAGATGATGCCGTCATCATCAAATTTTTTTTCTTGTTCCTGGGCTTTCACAACAATGGGAGCTGCTGCTGTGTTGGCGTTGACAAGCGCAACAGAGCAGAACGGCACGATACACCACAATAAAAGCAACTGCGAGCAACAGCCCGCGGTCTGGCTTTTCCCCAGCATACCTGAACATTCAAATTGCCAAGAGGATGGGAAAAAGACAGGCTAAAACTGTTGCCCACCTCATGGTCTCGGGTGGCATGAAATGTATTAGTCGTACATGATCAACTACCAAGGAGCGTTGGGTAATCTCCTTAACATGTTGCAGATAACTGGTGAAATTGACACAAACAGC

1-33 (reverse, 10:122069236-122069269, downstream Tmem5) 97.06% match

33-148 (reverse, 10:59768138-59768253, exon 8 Micu1) 100.00% match

GL261 fusion: Setd2-Rnf183

GTCGATCGCACGAGAT//GGGGGATTTCTACGATCCCGAGCACCCGACCCCTGAGTATGCTTTTGGACATGACTAAGACACCCCAGAGAAGACTAGAGAGAAACAGCTGCAGCCAACTCTGTTTTGAGTTCCAAAGTCATCCGAGGAAGGAAGAAGAAAGCCAGCCAGAGAGTGGACCAAGAGCTGGGGAGACCCATGCAAAAGAGGC//CAAAAACACACGGAGCTGGGGAGACCCATGCAAAAGAGGCCATTTCCAGATATAAAACTCCTGAAAATGGGGGCTGCTGCCGTTGTTCCTGTTACAAAGAAAAAAGAGGCACTGAGACGAGTACCAGACTTGGGCAATTGCTGATAGAAACCCACGGACCTGGGTCCCCTCACGCATAAAACACCAAATTTCTCCCAGAACCAAAGAAAGACTGGCGCGTAGGCTGTCTCCCCTCATGTGGTCACGGGGACACAGTCAAGATTCAGCACCTGATGAACCACCACGGTGAGCTCTGCAGCATCGCCACAGCGGCCCCAATACCGTAATTTTCCGAACCGATGAACAATCCCTGAATATTTTTGCCTAGAACCTGGTCGCCTATT

1-39 (forward, 9:110532717-110532755, exon 1 Setd2) 100.00% match

40-108 (reverse, 4:62438391-62438459, upstream Rnf183) 100.00% match

109-190 (reverse, 4:62434553-62434634, exon 2 within 5’-UTR, Rnf183) 100.00% match

GL261 fusion: Tmc1-Rfx3

TCTAGCCCGGACAAGCTGGACCCGGTTC//ATGCCGCCTCTTTCGGAAAACTAATAAAGTCCATTTTTATGGGGCTACGAACTAGGAGATTAGGCACTAGGTCAGGCTGGCCACCTGAATCTGTAACAGGTGAGTCGGGAAACAAAACAGAATAGCCTAGGAAAAGACACTACGG//GCTTGATTCTGTTTTGTTTCCCACTCACCTGATTACAAATTCGGTAGCCAGCCTGACCTAGTGCCTAATCTCCTAGTTCGTAGCCCCATAAAAATGGACCTTATTAGTTTTCGAAAGAGGCGGCATTGACCGGGTCCAGCTTGTGCTCCTGGCAGTGTCGAAGGTAGTGGTTGTACGCGTGCCACGTCAGAGCTGACTGACGTCAAGCCGCCATCCAAACAATCAGATTCACCGCACATCACAAGAAAGACAGATCACCTGCCGGCCCCAGATGATTGCCTTCCCATGCGGGAAAAAAAAAGATAAATCTCATAT

1-72 (reverse, 19:27830609-27830680, exon 7 Rfx3) 98.61% match

67-145 (reverse, 19:20985597-20985674, upstream Tmc1) 98.73% match

GL261 fusion: Tmem2-Rfx3

GCGGACTCGATCTTAGC//ATGATCATCACACGGGATGAATACCCATCCCACCCAATGGTGCTTCGGGGTATCAACCAGAGGGCCATCTCTCCACAGTACCAGCCTGTTGTCATGCTGGAGAAGGGCTACACCATCCACTGGAATGGTCCAGCGCCACGAACCACTTTCTTGTACCTCGTGAACTTCAACAAAGATTTTTATCAGAGGAATGTAGACTGGAATCTGCAATGGTTCTCAAAATCCCAAAGTGAGACCATCATGCAGACTTCAGAGACGGGTTCAGACACAGGTTCGACAGTGACTCTGCAGACGTCTGTGGCTAGCCAAGCAGCAGTGCCTACACAGGTGGTACAGCAAGTGCCAGTGCAGCAGCAGGTGCAGCAGGTACAGACAGTTCAGCAGGTCCAACATGTCTACCCAGCTCAGGTGCAGTATGTGGAAGGAAGTGATACTGTCTATACCAATGGAGCAATCCGAACAACAACTTATCCCTACACAGAAACACAGATGTACAGCCAAAACACTGGAGGAAATTACTTTGATACTCAAGGAAGTTCTGCCCAGGTGACAACTGTGGTGTCCTCCCACAGTATGGTGGGTACTGGTGGGATTCAGATGGGCGTCACAGGAGGACAACTCATCAGCAGCTCGGGAGGAACCTATTTG//ATCGGCAATTCAAATGGAGAACTCTGGTCACTCAGTGACACACACAACTCGGGCCTCCCCAGCAACATTGAAAATGGGGGATCCTTCAAATAAGGTTCCCTCCCCGAGCTGCTAATGAGTTGTCTCCCGTGACGCACATCTTAATCCCATCAGTACCCACCATACTGTGGGATGACACCACAGTTGTCACCTGGGCAGAACTTACTGAGTATCAAAGTAATTTCCTCCAGTGTTTTGGCTGTACTCTGCTGTTTCTGTGTAGGGATAGTTGTTGATTCGGATTGCTCATTGGTATAGACGCTATCATTCTTCCATCACTGCTCGAGATGGGGCAAACTGTTGGGACTGGCTGAAATGTCGTACTCGTCGCCGGCGTCCTCTGGGACTTGGGGCACACCGGTAGGACTGCTGCTCGGTCGCCTGACGCTGCTAGTACTGCGACTTTGTTGACCGTTCTATCTGCTGATGTTTCACTTTTGGCATTTGAACATTGCCATCAGCTCAATTCCTCTAATCTTGTCTGAATCAGGTCAGAGGGTTCTGCCTCGCGACTTGTGAAGGTACCCTCCCGGACCCCGCCTATATGCAAATGGCCTCCTGTGTATCACACATCTGAACGGATTCT

1-173 (forward, 19:21835454-21835626, exon 19 Tmem2) 100.00% match

174-232 (reverse, 19:27923208-27923266, exon 2 Rfx3) 100.00% match

233-357 (reverse, 19:27900779-27900903, exon 3 Rfx3) 100.00% match

358-455 (reverse, 19:27867513-27867610, exon 4 Rfx3) 100.00% match

456-648 (reverse, 19:27849777-27849969, exon 5 Rfx3) 100.00% match

GL261 fusion: Trim2-Gipc2

ACTTCTGCAT//GCTAGCGTCACCTGATCATTGTGACAACCGAGAGCTCTCCCAGATCTCAGCTATTTCCTTAGAGGTTGGTACCACCCACCCCTGCCCTAGTCCTGTTACTTGGCTCCCGGCTCCTGCAGGCGGTGCTTCACACCGGGAGGACAGAAGACCATCTCAGCTGAGTTTAGGCTTCTGGATCAATCTTACGGAATGCGTTGGTTCTTCTCTAGATGTTGAAAATAGAACTCCAGGCAAGCACTGAGCCACAGTCCTGGCTCTGATCCACTCCCATCAAAAACCTGGATCCTGCTGTTCCCCCAGTCAGCCACAATGATGTTCCCATTTGAGTCCACGGCCACACCTGTTGGTGCATTAAACTGCCCATTTCCTTCTCCATTGGAGCCAAACTTCAACATGAATTCTCCTTCCTGATTAAATACC//TTGCAGAGTGATAA

1-258 (reverse, 3:152147366-152147623, intron 1 Gipc2) 100.00% match

277-420 (forward, 3:84167650-84167793, exon 11 Trim2) 100.00% match

**SUPPLEMENTARY FIGURES**

**Supplementary Figure S2:** Gene expression level of *CD8A* (a), CD8 T-cell inhibitory receptors *PD-1* (b), *Tim-3* (c), *LAG-3* (d), *CTLA4* (e), and inhibitory ligand *PD-L1* (f) in the St. Jude cohort. Expression values are reported as fragments per kilobase of transcript per million mapped reads (FPKM). (PD-1=programmed death-1, Tim-3=T cell immunoglobulin and mucin protein 3, LAG-3=lymphocyte-activation gene 3, CTLA4=cytotoxic T-lymphocyte associated antigen-4, PD-L1=programmed death ligand 1, OS=osteosarcoma tumours, OB=osteoblasts controls).


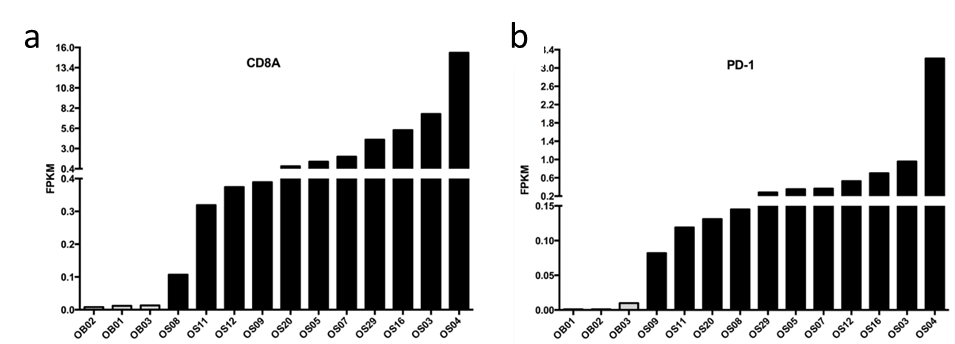

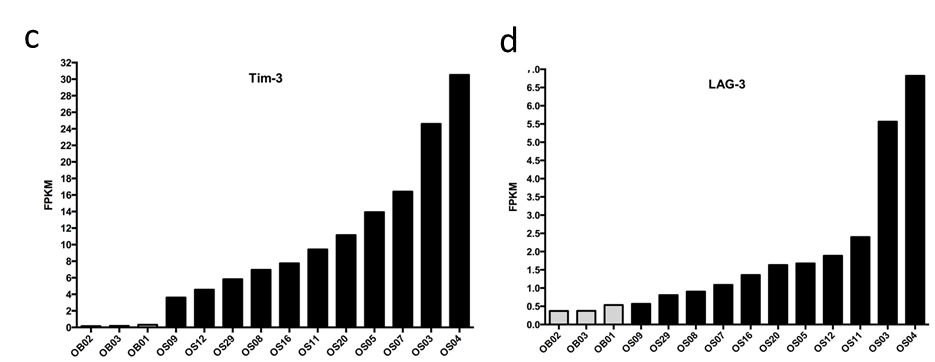


**
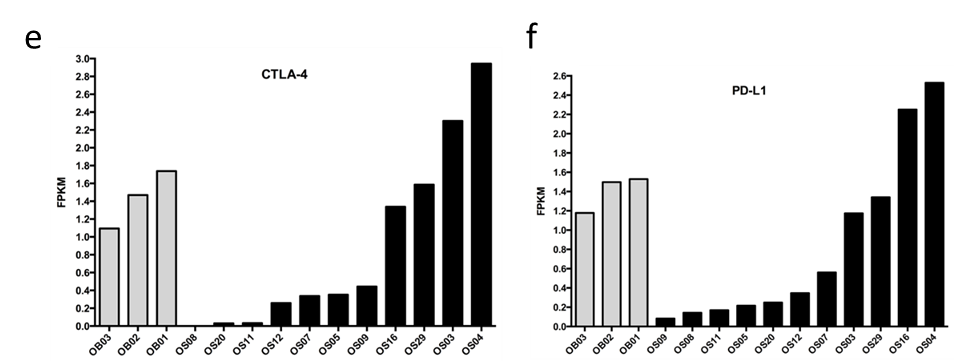
**

**Supplementary Figure S3:** Gene expression level of several antigen processing and presenting machinery components: *HLA-A* (a), *HLA-B* (b), *HLA-C* (c), *B2M* (d), *TAP1* (e), *TAP2* (f), *TAPBP* (g), *PSMB5* (h), *PSMB6* (i), *PSMB7* (j), *PSMB8* (k), *PSMB9* (l), *PSMB10* (m) in the St. Jude cohort. Expression values are reported as FPKM for all genes with the exception of HLAs that are reported as RPKM. (*B2M*=β2-microglobulin, *TAP*=transporter associated with antigen processing, *TAPBP*=TAP binding protein, *PSMB*=proteasome subunit beta, OS=osteosarcoma tumours, OB=osteoblasts controls).

**
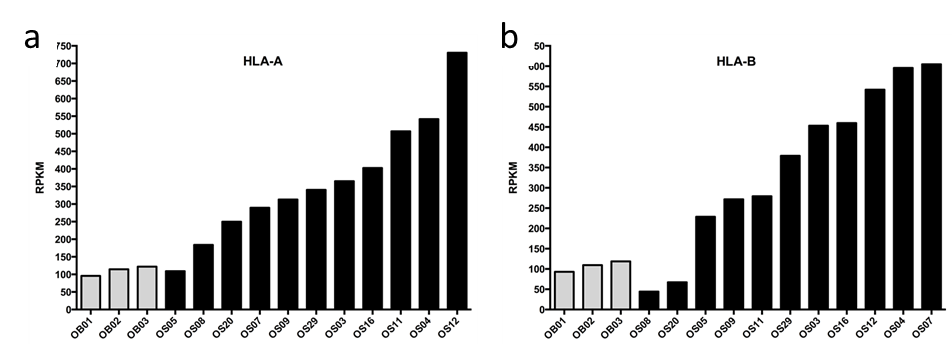
**

**
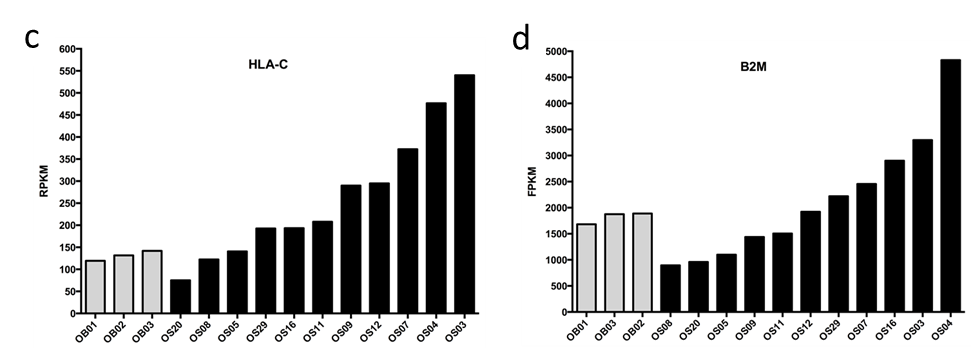
**

**
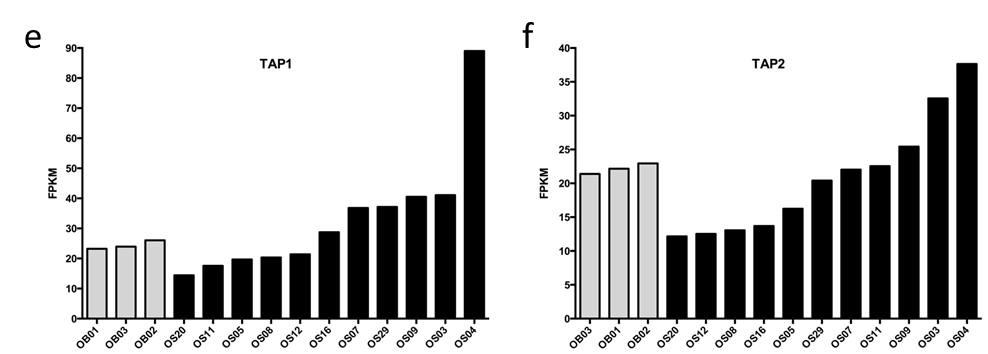
**

**
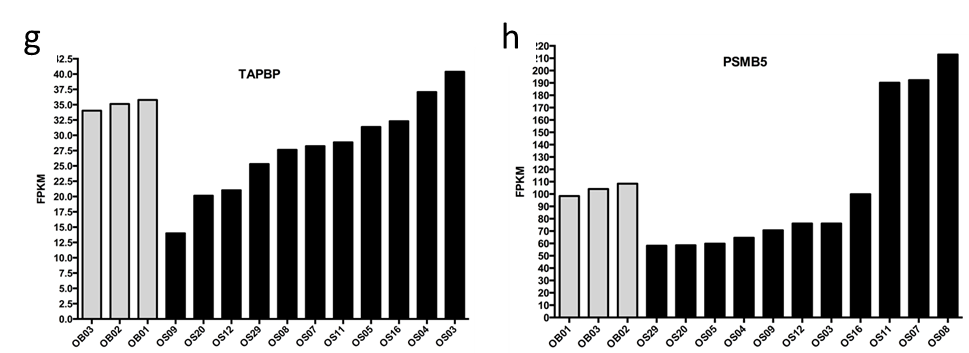
**

**
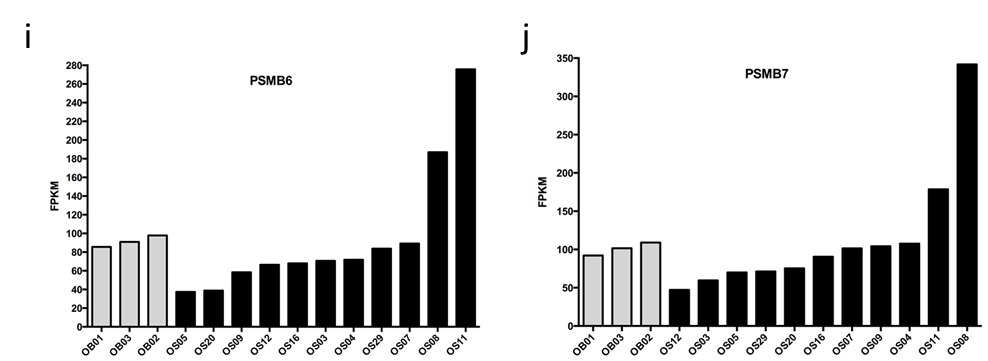
**

**
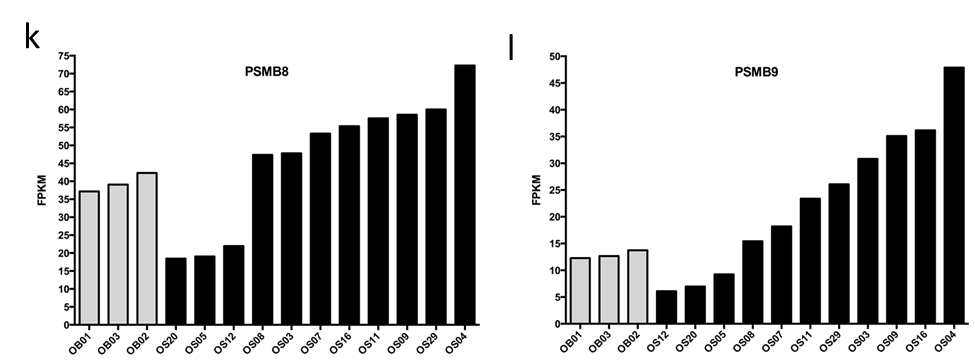
**

**
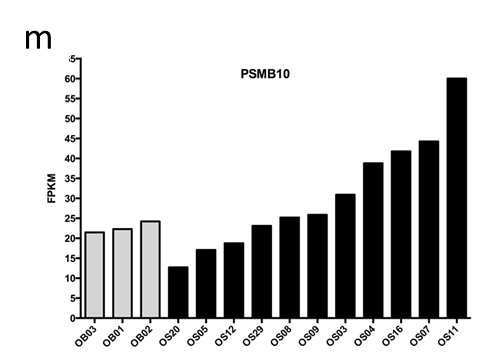
**

**Supplementary Figure S4:** Gene expression level of *CD8A* (a), CD8 T-cell inhibitory receptors *PD-1* (b), *Tim-3* (c), *LAG-3* (d), *CTLA4* (e), and inhibitory ligand *PD-L1* (f) in the Perry et al. 2014 dataset. Expression values are reported as fragments per kilobase of transcript per million mapped reads (FPKM). (*PD-1*=programmed death-1, *Tim-3*=T cell immunoglobulin and mucin protein 3, *LAG-3*=lymphocyte-activation gene 3, *CTLA4*=cytotoxic T-lymphocyte associated antigen-4, *PD-L1*=programmed death ligand 1, BZ=osteosarcoma tumours, OB=osteoblasts controls).

**
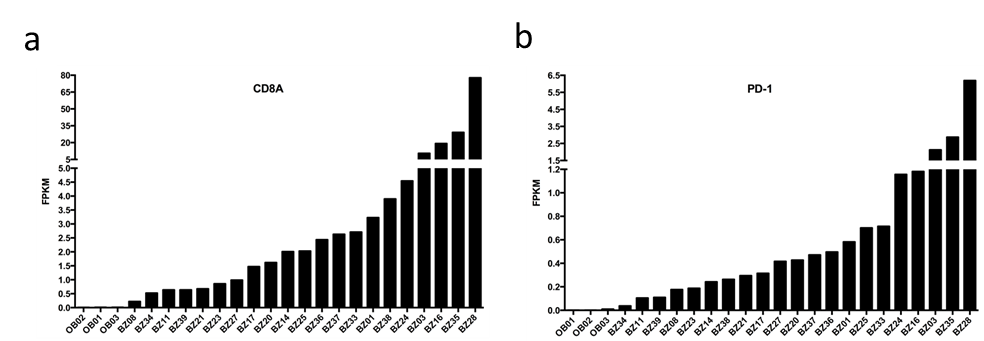
**

**
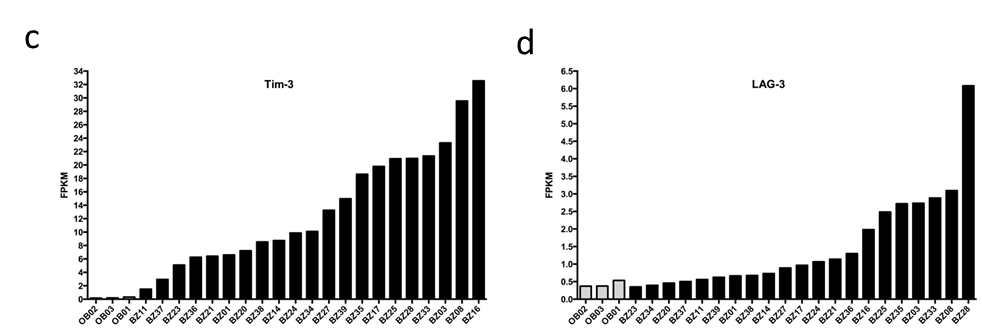
**

**
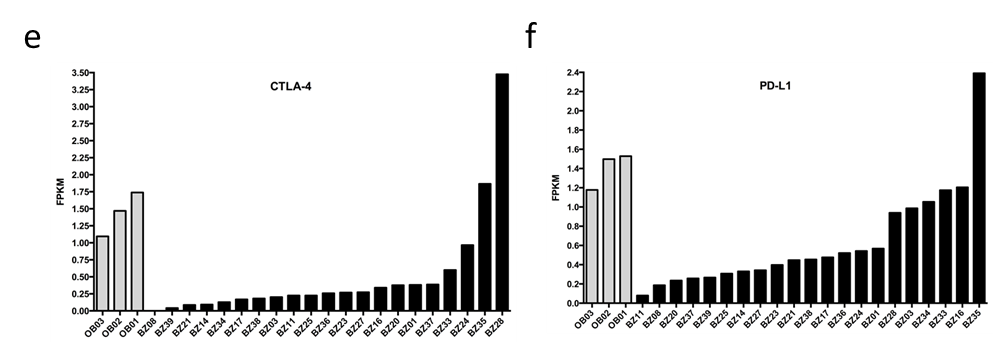
**

**Supplementary Figure S5:** Gene expression level of several antigen processing and presenting machinery components: *HLA-A* (a), *HLA-B* (b), *HLA-C* (c), *B2M* (d), *TAP1* (e), *TAP2* (f), *TAPBP* (g), *PSMB5* (h), *PSMB6* (i), *PSMB7* (j), *PSMB8* (k), *PSMB9* (l), *PSMB10* (m) in the Perry et al. 2014 dataset. Expression values are reported as FPKM for all genes with the exception of HLAs that are reported as RPKM. (*B2M*=β2-microglobulin, *TAP*=transporter associated with antigen processing, *TAPBP*=TAP binding protein, *PSMB*=proteasome subunit beta, BZ=osteosarcoma tumours, OB=osteoblasts controls).

**
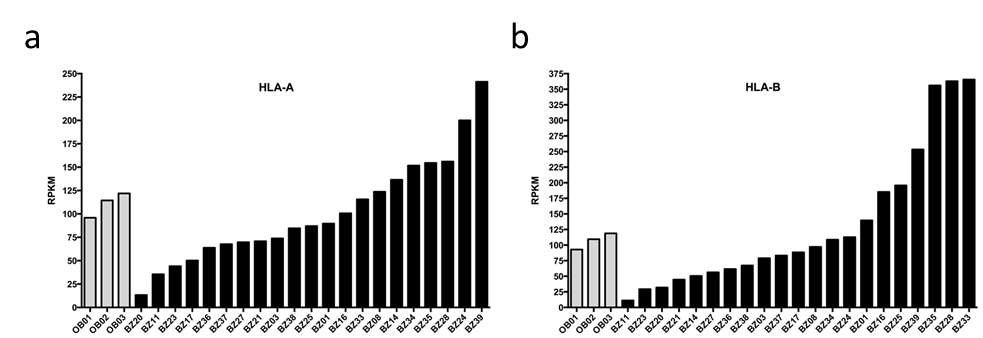

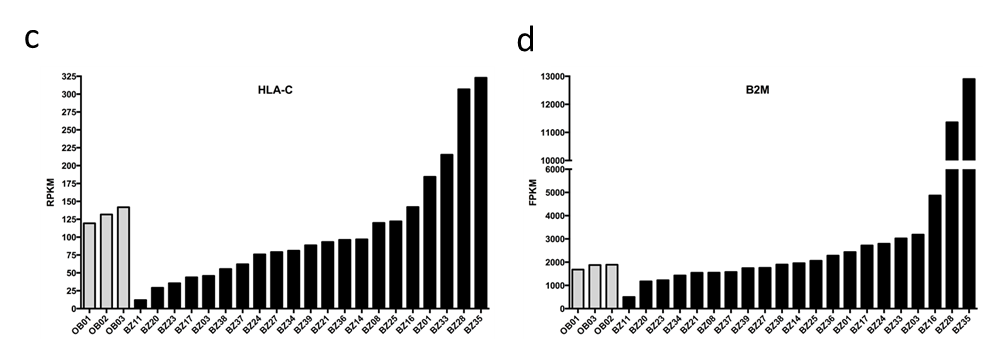
**

**
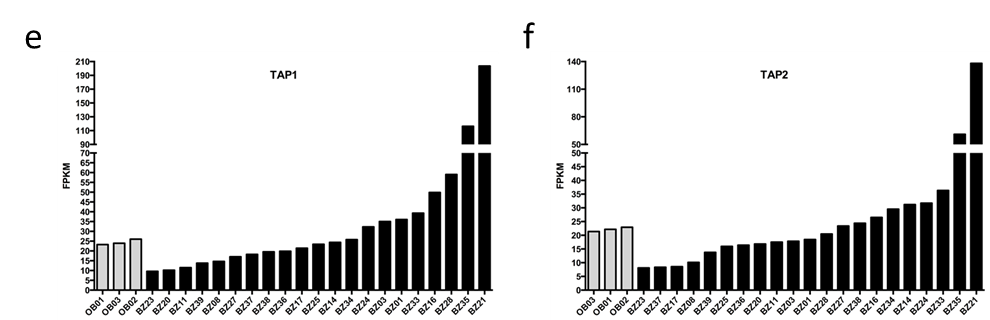
**

**
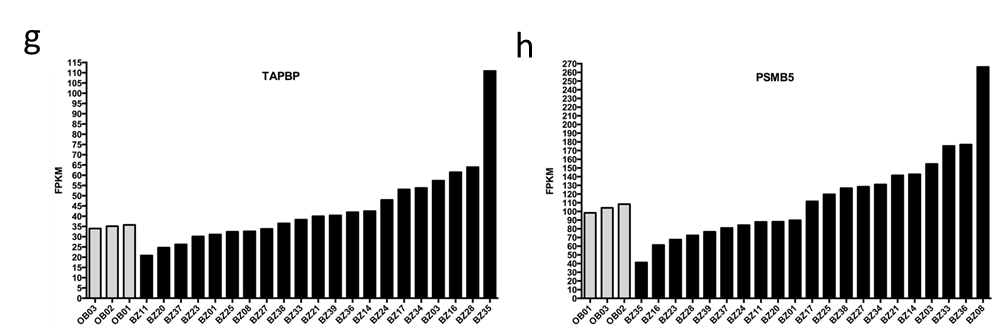
**

**
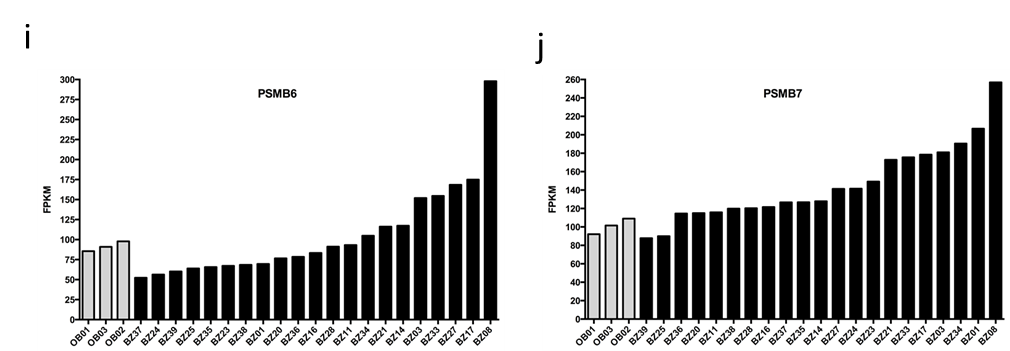
**

**
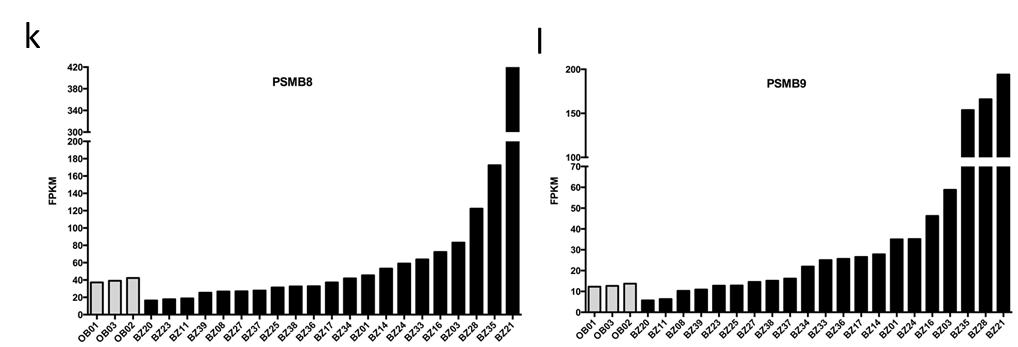
**

**
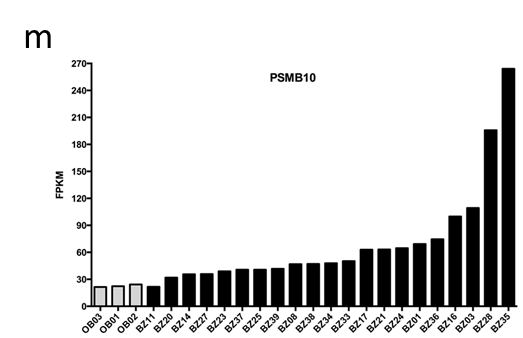
**

**Description of Supplementary Tables**

**Supplementary Table S1:** Fusion fractions calculations, including the source of the samples analysed.

**Supplementary Table S2:** Full name of the St. Jude samples.

**Supplementary Table S3:** Quality of RNA-seq mapping of St. Jude cohort and Perry *et al*. 2014 samples.

**Supplementary Table S4:** Detailed analysis of the INTER-chromosomal fusion detected by deFuse in the St. Jude samples, showing the 12 bp sequences on either side of the fusion breakpoint and used by the deFuse-Trinity comparison program to predict the presence of protein producing transcripts. Includes a description of novel proteins detected.

**Supplementary Table S5:** Detailed analysis of the INTRA-chromosomal fusion detected by deFuse in the St. Jude samples, showing the 12 bp sequences on either side of the fusion breakpoint and used by the deFuse-Trinity comparison program to predict the presence of protein producing transcripts. Includes a description of novel proteins detected.

**Supplementary Table S6:** List of predicted protein modifying fusion found in LLC1 and GL261 samples and the primers used to verify the presence of the fusion.

**Supplementary Table S7:** List of TICs detected by deFuse in 3 or more of the St. Jude samples and the samples in which they were verified in the Trinity data.

**Supplementary Table S8:** Calculation of the ratio of TMEM165-CLOCK reads to CLOCK reads in the St. Jude samples.

**Supplementary Table S9:** Output from the deFuse-Trinity comparison program for the human OS samples in the Perry et al. 2014 dataset, the canine OS and osteoblast samples in the Scott submitted dataset, the human OS samples St. Jude cohort, and the human osteoblast samples (sample names and sources described in Supplementary Table S1). INTER- and INTRA-chromosomal fusions used in the calculated of the fusion fraction are highlighted in green, while duplicates and those associated with *SRGAP2* are highlighted in red.

**Supplementary Table S10:** Search sequences used to determine the prevalence of the fusions in the common fusion table (Table 2).

**Supplementary Table S11:** Predicted CD8 T-cell neoepitopes with strong binding to patient-specific HLAs identified using NetMHC 4.0 and NetMHCpan 3.0 epitope prediction algorithms in the St. Jude cohort. (I_pos (Ip)=position of the insertion, if any. I_len (Il)=length of the insertion. D_pos (Gp)=position of the deletion, if any. D_len (Gl)=length of the deletion. Core=the minimal 9aa binding core directly in contact with the MHC. iCore=interaction core; the binding core including eventual insertions or deletions.)

**Supplementary Table S12:** Predicted CD8 T-cell neoepitopes with strong binding to patient-specific HLAs identified using NetMHC 4.0 and NetMHCpan 3.0 epitope prediction algorithms in the Perry *et al.* 2014 dataset. (I_pos (Ip)=position of the insertion, if any. I_len (Il)=length of the insertion. D_pos (Gp)=position of the deletion, if any. D_len (Gl)=length of the deletion. Core=the minimal 9aa binding core directly in contact with the MHC. iCore=interaction core; the binding core including eventual insertions or deletions.)

**Supplementary Table S13:** HLA typing information for osteosarcoma patients. Two alleles of HLA-A, B, C genes for each patient were predicted at four-digit resolution using the OptiType algorithm.

**Supplementary Table S14:** Predicted CD8 T-cell neoepitopes with strong binding to patient-specific HLAs in *TMEM165-CLOCK* TIC, identified using NetMHCpan 3.0 epitope prediction algorithm in the St. Jude cohort. (Ip=position of the insertion, if any. Il=length of the insertion. Gp=position of the deletion, if any. Gl=length of the deletion. Core=the minimal 9aa binding core directly in contact with the MHC. Icore=interaction core; the binding core including eventual insertions or deletions.)

**References**

1. McPherson, A. *et al.* deFuse: an algorithm for gene fusion discovery in tumor RNA-Seq data. *PLoS Comput Biol* **7**, e1001138 (2011).

2. Blankenberg, D. *et al.* Galaxy: a web-based genome analysis tool for experimentalists. *Curr Protoc Mol Biol* **Chapter 19**, Unit 19 10 1-21 (2010).

3. Giardine, B. *et al.* Galaxy: a platform for interactive large-scale genome analysis. *Genome Res* **15**, 1451-5 (2005).

4. Goecks, J., Nekrutenko, A., Taylor, J. & Galaxy, T. Galaxy: a comprehensive approach for supporting accessible, reproducible, and transparent computational research in the life sciences. *Genome Biol* **11**, R86 (2010).

5. Carrara, M. *et al.* State of art fusion-finder algorithms are suitable to detect transcription-induced chimeras in normal tissues? *BMC Bioinformatics* **14 Suppl 7**, S2 (2013).

6. Zhang, J. *et al.* INTEGRATE: gene fusion discovery using whole genome and transcriptome data. *Genome Res* **26**, 108-18 (2016).

7. Grabherr, M.G. *et al.* Full-length transcriptome assembly from RNA-Seq data without a reference genome. *Nat Biotechnol* **29**, 644-52 (2011).

8. Cunningham, F. *et al.* Ensembl 2015. *Nucleic Acids Res* (2014).
